# Horses discriminate human body odors between fear and joy contexts in a habituation-discrimination protocol

**DOI:** 10.1038/s41598-023-30119-8

**Published:** 2023-02-25

**Authors:** Plotine Jardat, Alexandra Destrez, Fabrice Damon, Zoé Menard--Peroy, Céline Parias, Philippe Barrière, Matthieu Keller, Ludovic Calandreau, Léa Lansade

**Affiliations:** 1 CNRS, IFCE, INRAE, Université de Tours, PRC, F-37380 Nouzilly, France; 2 Developmental Ethology and Cognitive Psychology Laboratory, Centre des Sciences du Goût et de l’Alimentation, Institut Agro Dijon, CNRS, Université de Bourgogne-Franche-Comté, Inrae, Dijon, France; 3 Development of Olfactory Communication and Cognition Laboratory, Centre des Sciences du Goût et de l’Alimentation, Institut Agro Dijon, CNRS, Université de Bourgogne-Franche-Comté, Inrae, Dijon, France; 4 UEPAO, INRAE, F-37380 Nouzilly, France

**Keywords:** Ecology, Neuroscience, Zoology

## Abstract

Animals are widely believed to sense human emotions through smell. Chemoreception is the most primitive and ubiquitous sense, and brain regions responsible for processing smells are among the oldest structures in mammalian evolution. Thus, chemosignals might be involved in interspecies communication. The communication of emotions is essential for social interactions, but very few studies have clearly shown that animals can sense human emotions through smell. We used a habituation-discrimination protocol to test whether horses can discriminate between human odors produced while feeling fear *vs.* joy. Horses were presented with sweat odors of humans who reported feeling fear or joy while watching a horror movie or a comedy, respectively. A first odor was presented twice in successive trials (habituation), and then, the same odor and a novel odor were presented simultaneously (discrimination). The two odors were from the same human in the fear or joy condition; the experimenter and the observer were blinded to the condition. Horses sniffed the novel odor longer than the repeated odor, indicating they discriminated between human odors produced in fear and joy contexts. Moreover, differences in habituation speed and asymmetric nostril use according to odor suggest differences in the emotional processing of the two odors.

## Introduction

Animals are widely believed to sense human emotions through smell. Chemoreception is the most primitive and ubiquitous sense, and brain regions responsible for processing smells are among the oldest structures in mammalian evolution^1^. Thus, chemosignals might be involved in interspecies communication, including emotional communication. An emotion is defined as “an intense but short-living affective response to an event”^2^, and the expression and perception of emotions play an essential role in the regulation of social interactions in mammals^3^, including human-animal interactions. In the last two decades research on the sociocognitive capacities of domestic mammals associated with human-animal interactions has increased, providing insight into how animals perceive our emotions^4^.

Domestic mammals have been shown to perceive human emotions through several sensory channels. For example, horses, dogs, cats and goats react to the emotional facial expressions of humans^5–9^. Horses, dogs and cats also perceive human emotions in vocalizations^10–13^. Moreover, cross-modal experiments have shown that horses, dogs and cats can integrate visual and vocal stimuli of humans expressing anger and joy, indicating that these species have multimodal mental representations of these emotions (i.e., they have mental representations of human emotions that combine visual and vocal features^12–15^). In addition, these species seem sensitive to the emotional valence of visual and vocal expressions. Cats and goats showed a preference for expressions of joy rather than anger^5,6^, and the behavioral and physiological reactions of horses, dogs and cats to expressions of anger were similar to those observed when these animals experience negative emotions themselves^4,16^. For instance, horses showed an increase in heart rates following visual presentation of angry faces compared to happy faces^8^. In dogs, visual and vocal signals of human fear also seemed to provoke behavioral and physiological reactions^9,10^. The processing of human fear by domestic mammals is unclear as research on the perception of human emotions by these species has mostly focused on anger and joy or happiness^4^, except for a few studies on other emotions^17,18^. Moreover, very few studies have investigated the olfactory perception of human emotions by domestic mammals, although olfaction is a dominant sense for most mammals, including horses^19,20^.

The perception of conspecific odors has been documented among domestic mammals. For example, horses differentiated between samples bearing the odor of unfamiliar conspecifics in a habituation-discrimination test; these samples were obtained by rubbing a piece of material on the coats of conspecifics^21^. Habituation-discrimination tests involve presenting an odor twice in successive trials (habituation phase), and then presenting the same odor and a novel odor simultaneously (discrimination phase). Horse sniffing duration is expected to decrease during the habituation phase, and to be higher for the novel sample than the repeated sample during the discrimination phase if they are able to discriminate between the two odors. Preference tests can also be used to assess olfactory perception; for example horses sniffed the odor of defecations produced by conspecifics who directed more aggression towards them for longer than defecations from other conspecifics in their group^22^. Additionally, heifers and pigs preferred to eat from a dispenser bearing the odor of urine from an unstressed conspecific rather than a stressed conspecific^23,24^. Dogs also showed more stress-related behaviors when sniffing conspecific body odors produced during isolation, a stress-inducing situation, rather than during play^25^. These findings highlight the influence of the emotional state of the emitter on the response of the receiver upon sniffing the olfactory cues.

Beyond this sensitivity to conspecific odors, a few studies have reported that domestic mammals are sensitive to human odors, including emotional body odors. Emotional information, such as fear and happiness, is conveyed by chemosignals produced in the sweat of humans^26,27^. Apocrine sweat glands in the armpit are thought to release compounds of different natures and/or quantities in the sweat, such as adrenaline and androstadienone, according to the emotional valence of the emitter^28^. A few studies have suggested that domestic mammals can perceive our emotions through olfaction and are influenced by them. For example, cattle sniffed human sweat produced in a non-stressful context for longer than that produced in a stressful context^29^, and dogs can distinguish between human odors from baseline and psychological stress conditions^30^. Dogs also showed more stress-like behaviors^25^ and interacted less with an unfamiliar human^31,32^ after sniffing human sweat collected while watching a fear-inducing video rather than a joy-inducing video. Using the same type of stimuli, in a recent experiment horses were presented successively with odors of human happiness and fear in the presence of a familiar human^17^. Horses lifted their head and tended to touch the familiar person more when sniffing the odor from the fear condition compared to that from the joy condition, suggesting that they perceived fear in the first odor and reacted with a fear-related behavior. These results are promising and merit further elucidation with fully counterbalanced experiments (i.e., experiments in which the presentation order of stimuli and the collection of samples from participants is randomized) and incorporation with other behavioral evidence, such as laterality biases.

Indeed, the emotional response of domestic mammals to stimuli is revealed not only by specific behavioral responses, such as the ones described above (preferences and emotional behaviors), but also by detecting brain asymmetries in the processing of emotional expressions, as the brain hemispheres are differentially involved in emotional processing^33^. These asymmetries are assessed by observing the preferential use of an ear, eye or nostril, indicating the preferential involvement of the contralateral hemisphere (for vision and audition) or the ipsilateral hemisphere (for olfaction)^34^. In general, in domestic mammals, the right hemisphere is preferentially used for negative or intense stimuli whereas the left hemisphere is favored for positive or familiar stimuli^33^. For example, horses preferentially used their left ear (right hemisphere) to listen to a human growl and their right ear (left hemisphere) to listen to laughter^11^ or voices associated with a positive past experience^35^; additionally, horses preferentially looked at a human face expressing anger with their left eye (right hemisphere) rather than their right eye^8^. Regarding olfactory stimuli, horses have been observed to preferentially use their right nostril (right hemisphere) to sniff arousing or novel odors^36,37^.

The purpose of the present study was to further explore the olfactory perception of human emotions by horses. Specifically, we examined (1) whether horses can discriminate between human body odors produced in joy and fear conditions, and (2) whether horses showed any emotional reaction to these stimuli. We expected that horses would discriminate between the two human emotional odors, and that they would react differently to the odors from the joy and fear contexts.

## Methods

### Ethics statement

This study was reported in accordance with ARRIVE guidelines. It was approved by the Val de Loire Ethical Committee (CEEA VdL, Nouzilly, France, authorization number CE19—2022-1503-2). Animal care and experimental treatments complied with the French and European guidelines for the housing and care of animals used for scientific purposes (European Union Directive 2010/63/EU) and were performed under authorization and supervision of official veterinary services (agreement number F371752 delivered to the UEPAO animal facility by the veterinary service of the Département d’Indre et Loire, France). The horses lived in groups, were not food deprived during the experiment and did not undergo any invasive procedures.

Human participation in the experiment was carried out according to the Declaration of Helsinki and was approved by the Institutional Review Board of the University of Tours (authorization number 2022-029). All participants were fully informed about the general aims and methods of the study, and they provided written informed consent for the collection of samples as well as their use in the experiment.

### Horses

The study involved 30 Welsh mares (*Equus caballus*) aged 5.7 ± 2.3 years (mean ± *s.d.*) reared and living at the Animal Physiology Experimental Unit PAO (UEPAO, 37,380 Nouzilly, France, https://doi.org/10.15454/1.55738963217 28955E12), INRAE. These mares lived in groups in indoor stalls bedded with straw and had free access to an outdoor paddock. Hay and water were available ad libitum. These horses are used only for research purposes and are handled daily by humans. They have the opportunity to experience human emotions expressed by caregivers and researchers.

### Stimuli

The odor-collection method was adapted from previous studies on human body odor^28,29,38^. Human axillary sweat odor was collected from 24 adult participants (6 males and 18 females) who volunteered to take part in the experiment. They were recruited through an e-mail sent to all personnel of our research facility (660 people). Participants were asked to abstain from consuming products known to influence body odors (i.e., chili pepper, spices, blue cheese, onion, garlic, cabbage, tobacco, and alcohol), abstain from use of deodorant, perfume or scented lotion, and to wash with a perfume-free soap provided by the experimenters for 2 days before their sweat was collected. The morning before participants donated their sweat, they were asked to wash their armpits with clear water only. Given the small number of participants, the menstrual cycle of females was not discriminated.

Each participant took part in two individual sessions separated by at least 24 h, during which they watched a 20-min video meant to provoke fear or joy. The clip selected for the fear condition was an excerpt from the movie *Sinister*^39^ (judged as the most frightening horror movie in 2020—https://www.broadbandchoices.co.uk/features/science-of-scare). The clips selected for the joy condition were adapted from those used by de Groot et al.^28^: “Bare Necessities” from *The Jungle Book*, Kurt Kuene’s short movie *Validation*, and the dance scene from the film *The Intouchables*. The order of the conditions was chosen randomly for each participant and counterbalanced among participants (half of participants watched the fear-inducing video first and the other half watched the joy-inducing video first).

Immediately before watching the video, participants were required to wash their armpits with wet unscented cotton pads and dry them with an unscented paper towel. Then, they placed under each armpit two cotton pads (7.5 × 7.5 cm, Euromedis, Neuilly-sous-Clermont, France) that had been previously folded together and secured them in place with unscented surgical tape. They wore a provided unscented cotton t-shirt that was previously washed without detergent and did not wear any other clothes over it. After each session, participants placed the cotton pads and t-shirts in airtight sealed bags, which were stored in a freezer at − 20 °C for a maximum of six weeks. Participants rated their extent of fear and joy while watching the videos on 7-point Likert scales^28^.

Participants also indicated in a questionnaire whether they had thoroughly followed the dietary and hygienic instructions. The samples from nine participants had to be excluded from the experiment due to lack of compliance with the instructions. The samples from the remaining 15 participants were used as stimuli presented to horses.

### Procedure

The experiment took place over 2 weeks in January, 2022.

#### Sample preparation

One hour before the beginning of a habituation-discrimination test, the samples were thawed at room temperature in the airtight bags^17,28^. The stimuli were presented on 150-cm wooden sticks covered on one end by a single-use plastic bag that was changed for each odor presentation. Over the plastic bag was placed a piece of fabric (30 × 25 cm) from the armpits of a participant’s t-shirt, with an unfolded cotton pad from the same participant and same condition layered on top. For each horse, four wooden sticks were prepared with pieces of the t-shirt and pads from a single human participant; depending on which odor was used for the habituation phase, these sticks either consisted of three sticks with the odor from the fear condition and one with the odor from the joy condition, or vice versa. The samples were then covered with single-use plastic bags. To keep the samples warm despite winter weather, they were placed 15 cm from a heating lamp for 5 min before they were presented to the horse. For each condition (fear and joy), sweat was collected on four pads for each participant. This design enabled samples from each participant to be prepared for two horses (three pads from the joy condition and one from the fear condition for one horse and vice versa for the second horse). Thus, each set of four pads from one participant (either three pads from the joy condition and one from the fear condition, or vice versa) was sniffed by one horse.

#### Experimental setup

**Figure 1:**
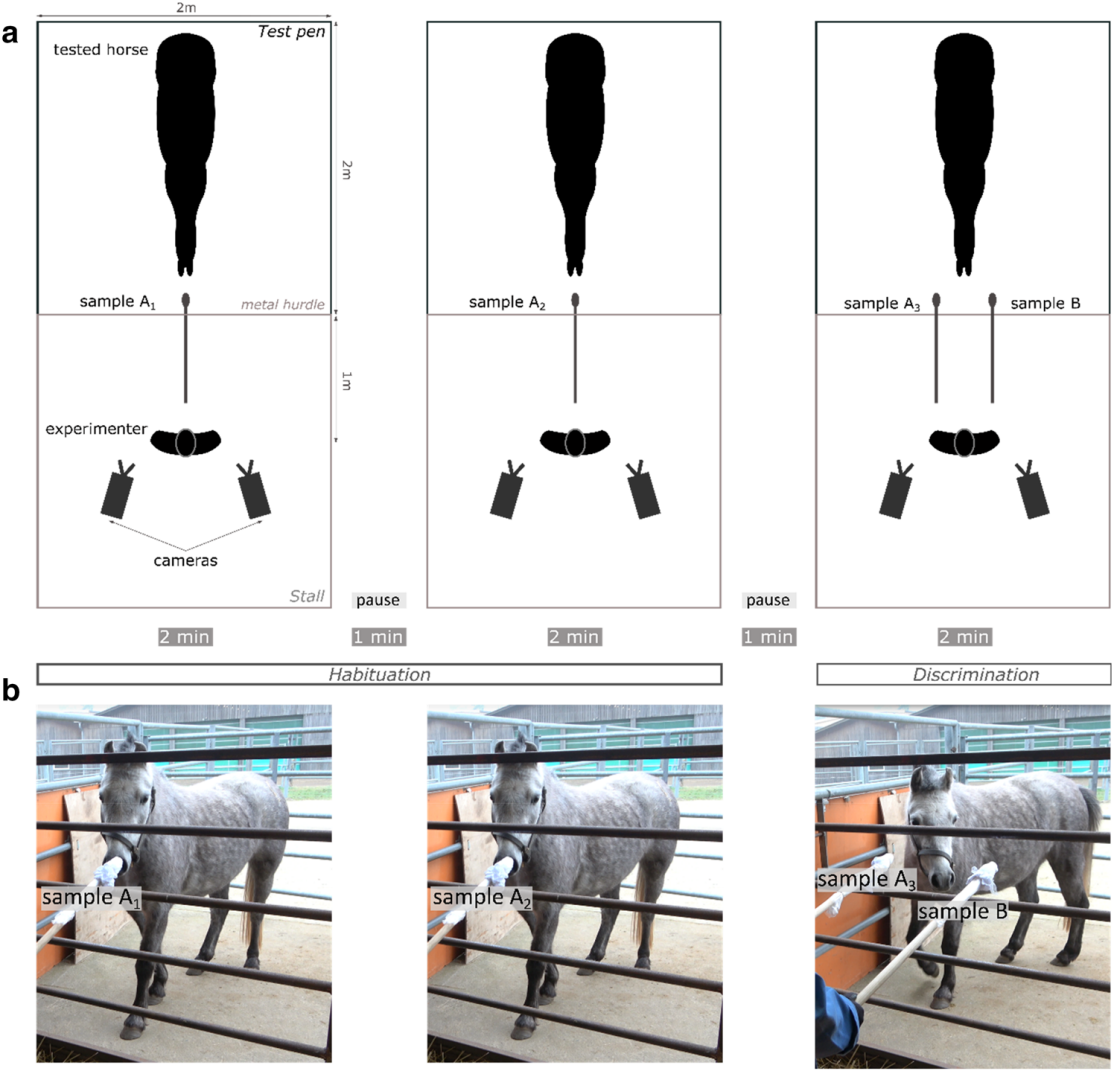
Habituation–discrimination protocol. (**a**) Schematic representation of the experimental set-up. (**b**) Photographs showing sample presentation. Photograph courtesy of Plotine Jardat.

The experiment took place in an outdoor pen (2 × 2 m) adjacent to an open stall where the experimenter stood (Fig. 1a). The wooden sticks bearing the odors were presented through a metal hurdle. Both the sticks and the hurdle were marked to standardize the stimulus presentation (the distance from the hurdle to the odor and the placements of the sticks on the hurdle were fixed). The experimenter stood 1 m from the hurdle, facing the horse. Two cameras were placed on to the left and right of the experimenter to film the behavior of the horse during the test. Two assistants who were not visible to the horse gave the samples to the experimenter at the appropriate time. The experimenter and assistants wore surgical masks hiding their facial expressions and did not wear any perfume. They were as immobile as possible and never looked directly at the horse. The experimenter looked strictly in front of them, with a 45° angle towards the ground. Importantly, the experimenter presenting the odors to the horse was blind to the odor condition: they did not take part in the preparation of the sticks and could not discriminate between them once prepared.

#### Familiarization

The familiarization phase began when the horse was released in the test pen. This phase lasted at least 30 s, during which the experimenter and the assistants quietly placed themselves. The test could then begin as soon as the horse was calm (not neighing, trying to escape the pen or circling). All horses met this criterion within two minutes.

#### Habituation-discrimination test

The test was a habituation-discrimination procedure adapted from^21^ and was comprised of two phases: habituation and discrimination (Fig. 1 and Supplementary Material—Video S1). During the habituation phase, a sample (A_1_) with odor A was presented to the horse at the center of the hurdle at a height of 1 m for two minutes; then, a one-minute interval elapsed, before a second sample (A_2_) with odor A was presented to the horse for two minutes in the same way. Another one-minute pause was observed between the habituation and discrimination phases. During the discrimination phase, two samples were presented simultaneously to the horse, 50 cm apart; one of these samples (A_3_) carried odor A, the repeated odor, and one carried odor B, the novel odor. The two samples were presented at the same time and speed and were equidistant from the previous sample location (Fig. 1). Half the horses were presented with the odor from the joy condition as samples A_1_, A_2_ and A_3_ and the odor from the fear condition as sample B, and vice versa for the other half (Table 1). Moreover, during the discrimination phase the location (left or right) of odors A_3_ and B and of the odors from the fear and joy conditions were randomly distributed and counterbalanced among horses (Table 1). Throughout the test, if the horse started biting the cotton pad or catching it with her lips, the experimenter had to move the stick 5 cm to one side to prevent the horse from swallowing the sample, then the stick was returned to the initial location within 1 s.

**Table 1:** Randomization of stimuli presentation.

Group	Sample				Location of odor B	Number of horses
	A_1_	A_2_	A_3_	B		
F	Fear	Fear	Fear	Joy	Left	7
					Right	6
J	Joy	Joy	Joy	Fear	Left	6
					Right	6

Of the 30 horses that participated in the test, 5 did not sniff the sample and were excluded from further analysis; this table gives information for the 25 remaining horses (see “Statistical analysis”).

### Behavioral analysis

The recorded videos of the tests were analyzed using BORIS v. 7.12.2^40^ by a coder who was blind to the side of odor B and to the type of odor (fear or joy condition) of each stick.

**Figure 2:**
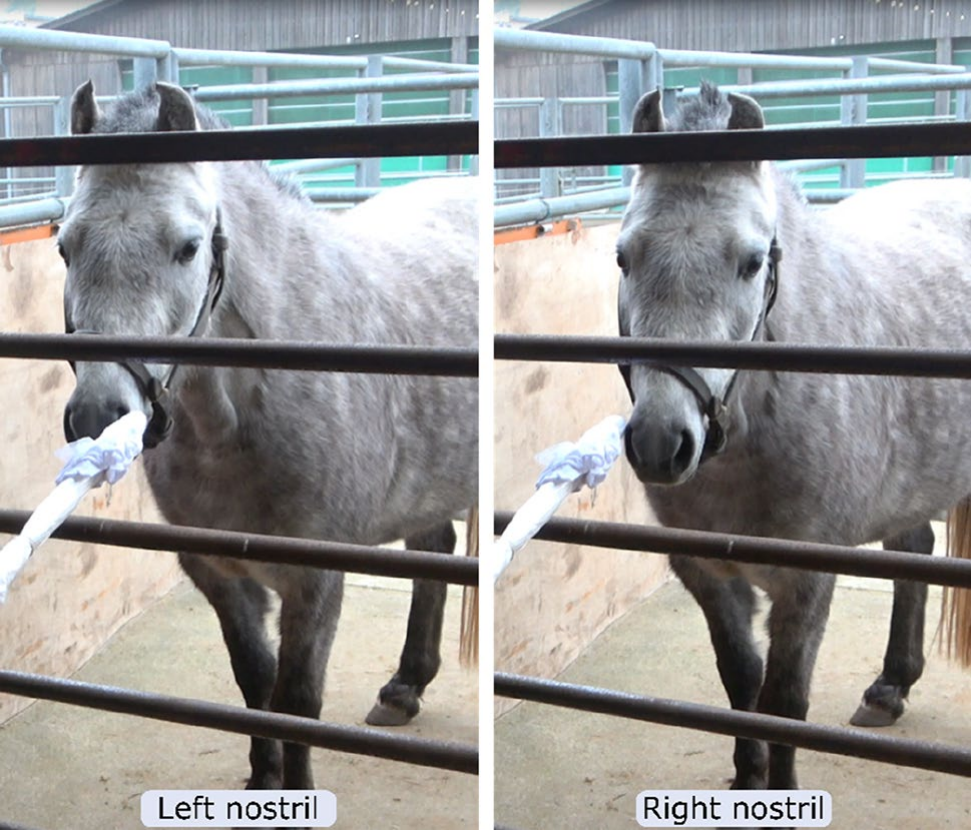
Example of a subject using her left or right nostril to sniff the sample. Photograph courtesy of Plotine Jardat.

The duration that each horse spent sniffing the samples was determined. Valid sniffing of the samples was defined as when the horse had its head turned towards a sample with a visible dilation of the nostrils and/or when its nose was 15 cm or less from a sample. Moreover, the preferential use of nostrils for sniffing the odors was analyzed; for each nostril, when it was directed towards the sample (touching or almost touching it) while the other was not directed towards the sample (Fig. 2), the number of nostril dilations and side of this nostril were noted. When both or neither nostril was directed to the sample, we considered that no nostril was being used preferentially. Flehmen responses (raising of the upper lip), defecations and neighs were also counted.

### Statistical analysis

All statistical analyses were performed using R 4.1.2^41^, and figures were generated using the *ggplot2* package^42^. The significance threshold was set at α ≤ 0.05 and tendencies were considered for p values ≤ 0.1.

To test whether the human participants experienced different emotions during the two videos, we compared the rating scores for ‘fearful’ and ‘joyful’ during the fear condition to those during the joy condition, using two-tailed paired permutation tests^28^ (*symmetry.test* function from the package *coin*^43^ with an exact distribution).

Of the 30 horses that participated in the test, 5 did not sniff any of the samples during the habituation phase (neither A_1_ nor A_2_) and were therefore excluded from further analysis. The duration sniffing the odors and the number of nostril dilations were explored with generalized linear mixed models (GLMM) from the package *glmmTMB*^44^, using Poisson distributions. The habituation and discrimination phases were analyzed individually. For both variables, an initial model was constructed for each phase, assessing the effect of the sample presented (A_1_ or A_2_, then A_3_ or B) and the effect of the group (J or F), representing which odor was presented during the habituation phase (i.e., the odor from the joy condition or fear condition, respectively), and their interaction. In addition, for the number of nostril dilations, the effect of the side of the nostril as well as its interaction with the other two factors was assessed. Horse identity was added as a random effect to account for individual variation in paired data, as each horse was presented with two samples in each phase. The variables included in each model were subjected to selection using a model comparison with two-tailed analysis of variance (ANOVA) with the null model and simpler models (without an interaction, then without each variable of interest). Distributions, within-group variance and homoscedasticity of the residuals were checked using the package *DHARMa*^45^ for each selected model, showing that the model assumptions were satisfied. When necessary, a post hoc test based on Tukey’s methods was performed with the package *emmeans*^46^.

The selected models are presented in Table 2 (see Table S1 for the detailed results of each ANOVA).

**Table 2:** Model selection results for each phase.

Response variable (y)	Model type	Family	Phase	Selected formula	χ^2^	AIC	DF	P value
Duration sniffing the odor	GLMM *n* = 25 × 4 odors	Poisson	Habituation	Sniffing duration ~ Sample * Group	10.41	397.43	1	0.0013
			Discrimination	Sniffing duration ~ Sample	11.66	360.89	1	0.0006
Number of nostril dilations			Habituation	Dilations ~ Side + Sample	5.97	413.67	1	0.015
			Discrimination	Dilations ~ Side * Sample	11.3	332.16	1	0.0008

To further analyze the first reaction of horses according to the type of odor presented, we focused on odor A_1_. We calculated a left-nostril bias index measuring the propensity to use the left nostril more than the right. This index was defined as L/(L + R), where L is the number of dilations of the left nostril, and R the number of dilations of the right nostril. This left-nostril bias could vary from 0 (indicating exclusive use of the right nostril) to 1 (indicating exclusive use of the left nostril); a score of 0.5 indicated equal use of both nostrils. Five horses did not show preferential nostril use for A_1_; we therefore were unable to calculate this index for these horses and excluded them from this analysis, focusing on the remaining twenty horses. We tested whether the horses used their left nostril more than the right nostril for each emotion (joy: *n* = 11, fear: *n* = 9), by comparing this index to 0.5 using one-tailed Wilcoxon tests (*wilcox.test* function with *mu* = 0.*5*).

Flehmen responses, defecations and neighs were exhibited by too few individuals to be considered in the statistical analysis (Flehmen responses: *n* = 1, defecations: *n* = 3, and neighs: *n* = 2).

## Results

### Participant emotions

**Figure 3:**
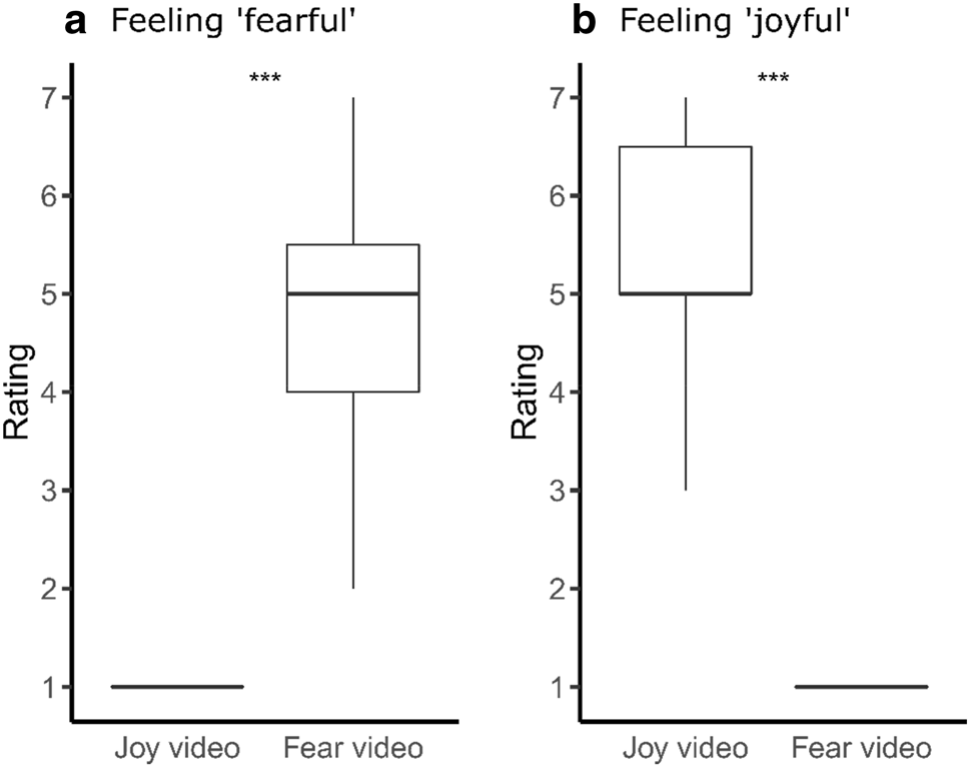
Emotion ratings reported by the participants after watching the fear- and joy-inducing videos. (**a**) Ratings for ‘fearful’. (**b**) Ratings for ‘joyful’. Boxplots show the median, first and third quartiles. Permutation tests, ***p ≤ 0.001.

Participant ratings of their emotions showed that they were significantly more joyful and less fearful after watching the joy-inducing video compared to the fear-inducing video (two-tailed paired permutation tests, *n* = 15; joyful: *Z* = 3.45, *p* < 0.001; fearful: *Z* = − 3.50, *p* < 0.001; Fig. 3).

### Habituation and discrimination of horses to the emotional odors

**Figure 4:**
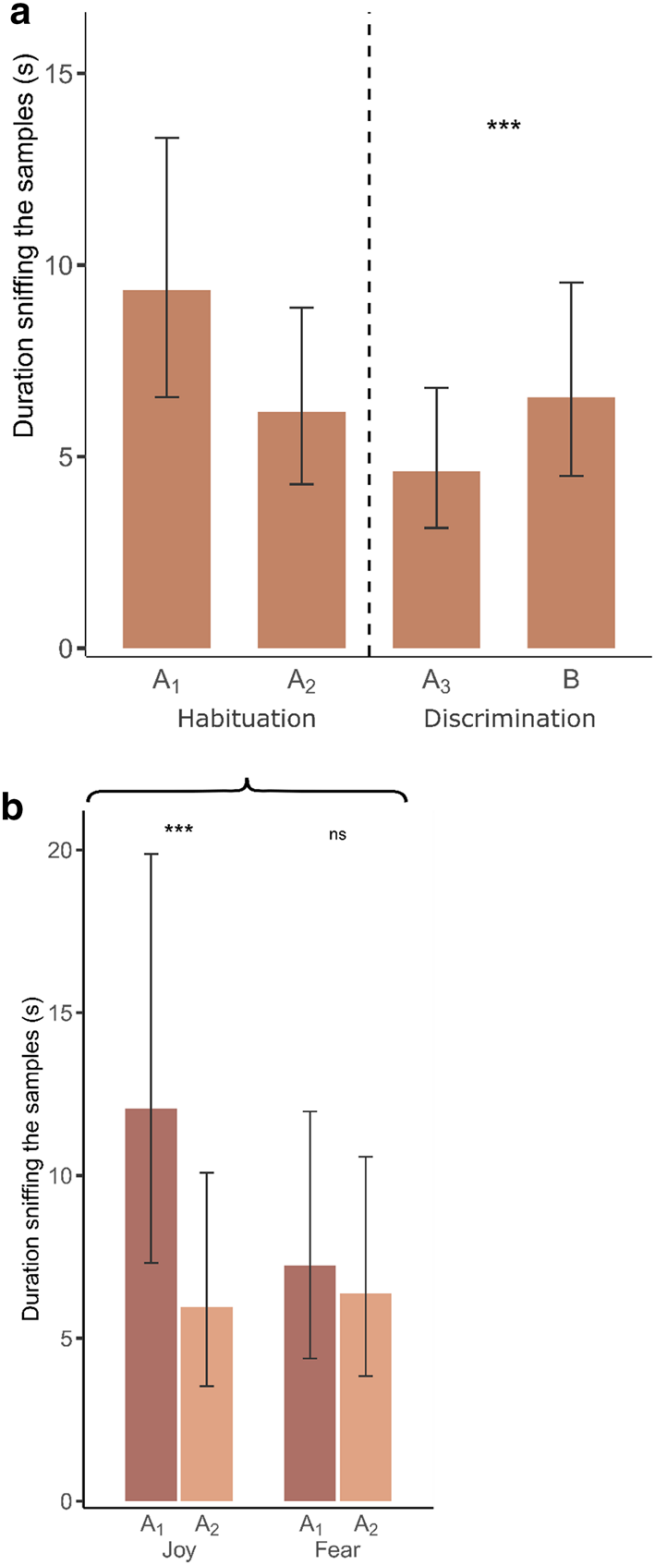
Habituation and discrimination to the samples. (**a**) Habituation and discrimination of all horses. (**b**) Habituation according to the type of odor (e.g., group). The graphs are extracted from the corresponding models presented in Table 2. The error bars represent the standard errors from the models. ***p ≤ 0.001, ns: *p* > 0.05.

The GLMMs showed that during the habituation phase, the time duration that horses sniffed the odors was affected by the sample x group interaction. Tukey’s post hoc tests revealed that horses from group J (i.e., the horses for which odor A was from the joy condition) sniffed A_2_ for a shorter time than A_1_, while it was not the case for horses from group F (i.e., the horses for which odor A was from the fear condition; Fig. 4, group J: *t* = 5.108, *p* < 0.0001; group F: *t* = 1.092, *p* = 0.28). During the discrimination phase, horses sniffed the novel odor (B) for significantly longer than the repeated odor (A_3_), regardless of their group (Fig. 4, *Z* = 3.388, *p* = 0.0007). Therefore, horses habituated to the presented odor when it was from the joy condition but not when it was from the fear condition; and discriminated between the new odor and the repeated odor in all cases.

### Preferential nostril use

**Figure 5:**
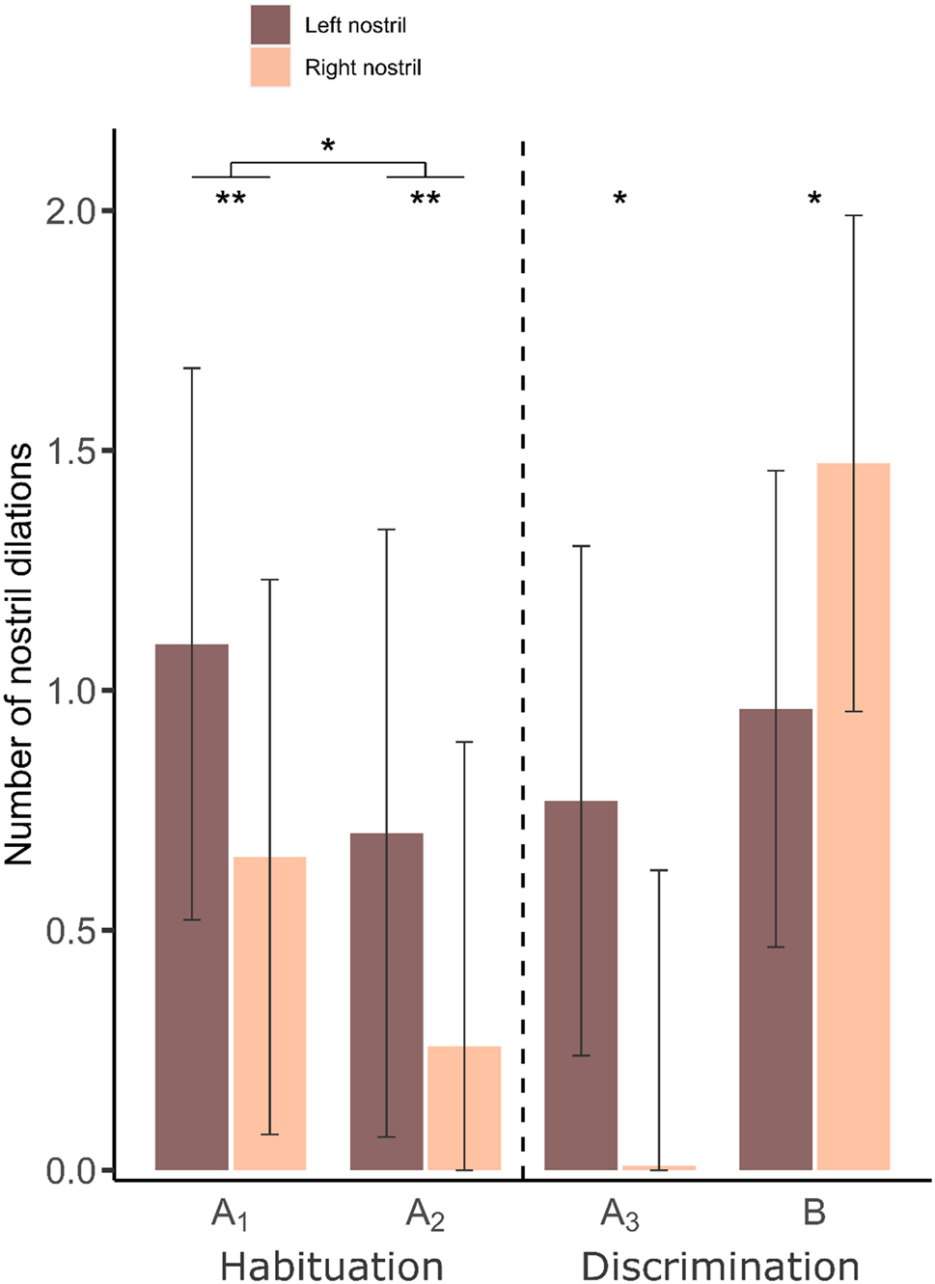
Differential use of the left and right nostrils when sniffing odors. The graphs are extracted from the corresponding models presented in Table 2 (see “Methods” section). The error bars represent the standard errors from the models. *p ≤ 0.01,**p ≤ 0.001.

The GLMMs showed that during the habituation phase, the number of nostril dilations close to the sample was affected by the side of the nostril and by the odor. The number of dilations decreased from A_1_ to A_2_ (Fig. 5, *Z* = − 2.48, *p* = 0.013), indicating that the number of nostril dilations was also influenced by habituation. Moreover, during the habituation phase horses preferentially used their left nostril to sniff the odors (Fig. 5, *Z* = − 3.03, *p* = 0.002). During the discrimination phase, the number of nostril dilations was affected by the side of the nostril side x odor interaction. Tukey’s post hoc tests revealed that horses used their left nostril more than their right nostril to sniff the repeated odor A_3_, whereas they used their right nostril more than their left nostril to sniff the novel odor B (Fig. 5, odor A_3_: *t* = 2.50, *p* = 0.014; odor B: *t* = − 2.21, *p* = 0.029). The group (i.e., whether the first presented odor was from the joy or from the fear condition; groups J and F, respectively) did not affect the number of nostril dilations, as it was not included in the selected models.

In addition, the left-nostril bias index was significantly higher than 0.5 for odor A_1_ when it was the odor from the joy condition but not when it was the odor from the fear condition (joy: *V* = 52, *p* = 0.049; fear: *V* = 23.5, *p* = 0.80), indicating that for A_1_ horses showed a significant left-nostril bias when sniffing joy but not when sniffing fear.

## Discussion

The main result of this study was that when presented with human odors from different emotional contexts in a habituation-discrimination test, horses sniffed the novel odor for longer than the repeated odor. This result is consistent with that of other habituation-discrimination tests^21,47^ and shows that horses are able to discriminate human body odors from two distinct emotional contexts when they are presented simultaneously. Moreover, when the repeated odor and the novel odor were presented simultaneously during the discrimination phase, horses preferentially used their left nostril to sniff the repeated odor and their right nostril to sniff the novel odor. This finding is consistent with the previous observation that horses preferentially used their right nostril for sniffing novel objects^37^, and it supplies additional evidence that horses differentiated between the two odors. These results from our experiment confirm the differential perception of human emotional odors by horses that had been suggested in the study of Sabiniewicz et al.^17^, reporting that horses presented with human emotional odors responded by lifting their head more and touching the familiar person more when sniffing the odor from the fear context compared to that from the joy context. If horses can perceive the emotional odors of humans, this raises the question of what compounds are the chemical basis for such interspecific communication. In humans, several compounds in sweat, such as adrenaline or androstadienone have been proposed as candidates that carry emotional information^28^, and recent findings support the notion of the signal-specificity of axillary odors for distinct emotional states^48^. The perception of human emotional information contained in sweat odors implies the existence of receptors for such compounds. These receptors could be present in horses, either as a result of domestication or by inheritance from a common mammalian ancestor. As several other species of domestic mammals seem to perceive these compounds (namely, dogs, cattle and mice^29,31,32^), the first hypothesis would entail multiple appearances of such receptors during the domestication of each of these species. However, olfaction is the most ancient and universal sense, and the cerebral structures that process odors evolved very early in mammals^1^. Therefore, the second hypothesis appears more parsimonious. The second hypothesis is also supported by the recent finding that humans could recognize fear and non-fear odors in horse sweat^49^, which could occur through existence of common chemical compounds and their receptors in all mammals.

During the habituation phase (samples A_1_ and A_2_), we detected a significant decrease in the duration sniffing the stimuli when the odor from the joy condition was presented; however, when the odor from the fear condition was presented, there was no significant difference in sniffing durations. The smell of fear could be more stimulating for horses than that of joy. Indeed, for humans the odor of fear appears more intense than that of joy: in a study, participants were more successful at distinguishing fear sweat from neutral sweat than happiness sweat from neutral sweat^50^. Moreover, studies have shown that the primitive role of olfactory signaling in humans seems to be the fight-or-flight response^26^, thus, the odor of fear could be alarming for horses, given their prey nature and reactivity to flight-triggering stimuli^51^. As a consequence, a longer duration or repeated presentations of the same stimulus may be necessary for horses to habituate to odors from a fear context compared to odors from a joy context. This differential processing of two emotional odors by horses suggests different perceptions of human body odors according to the emotional context of their production. Furthermore, when sniffing the first sample, horses exhibited a left-nostril bias for the odor from the joy condition but not for that from the fear condition. In mammals, nerve fibers from the left nostril project to the left hemisphere of the brain^33^; therefore, this result suggests a left hemisphere bias in horses when sniffing the joy-context odor during the first sample presentation. As a left hemisphere bias was previously observed in horses for listening to human voices associated with a positive past experience^35^, a human vocalization of happiness^11^, and vocalizations of familiar conspecifics^52^, this pattern suggests that horses perceived the joy-context odor as positive. Together, these results suggest that horses perceive human body odors from a positive and negative emotional context differently. This sensitivity to the emotional valence of human odors could lead to emotional reactions in horses, akin to the emotional contagion mechanism reported in humans^27,53^. Thus, horses’ emotions could also be influenced by those scented on humans as a consequence of either spontaneous responses to the chemical compounds or a learned association between odors and the situations in which they are encountered^1^.

In this study, we also observed that during the habituation phase, horses used their left nostril significantly more than their right nostril, suggesting that horses explored these human body odors with a left hemisphere bias. Such bias is usually observed when exploring positive or familiar stimuli in domestic mammals^33^; thus, these results indicate that the horses in this experiment perceived human body odors as positive or familiar stimuli, which can be explained by an overall positive relationship with humans. Moreover, horses used their right nostril significantly more than the left in the discrimination phase. It is possible that after recognizing in the habituation phase that the two samples were produced in the same emotional state by the same person, horses were somewhat surprised by the different emotional state they smelled in sample B. Indeed, other studies found the right hemisphere to be preferred for the evaluation of novel stimuli and situations that may request quick reactions^54–56^.

## Limitations of the study

To avoid multiplying the number of factors included in the analysis, only female horses were involved in this study. In humans, sex differences in the perception of emotional chemosignals have been established, with women showing better classification of a happiness odor than men^57^ and showing larger effects of olfactory-induced emotional contagion^27,58^. In dogs, sex differences have also been revealed, with females reacting more strongly to a human happiness odor^32^. However, stallions are more reactive to interspecific odors than mares and geldings^59^; therefore, it would be interesting to conduct experiments to assess sex differences in horses regarding the perception of human emotional odors. It would also be interesting to examine potential variations of horses’ response to human emotional odors according to their temperament, as gusto-olfactory sensitivity and fearfulness are part of the temperament traits of horses^60,61^. Further studies could also explore the influence of different stress levels of horses on the perception of human emotional odors, as higher stress levels were associated with higher olfactory sensitivity in humans^62^. Hormonal status of the receiver (horses) could play a role as well, considering that odor exploration seems to be influenced by reproductive status in mares^63^ and women^64^. Finally, further studies may consider including a control group (odors A vs A in the discrimination phase) to rule out any effects caused by a change in the number of samples between the habituation and discrimination phases.

## Conclusion

In this study, we showed in habituation-discrimination tests that horses can discriminate between human odors produced in a joy *vs*. fear context. Moreover, differences in habituation speed and asymmetric nostril use according to odor suggest a differential emotional processing of the two odors. This study adds olfaction to audition and vision as senses through which horses perceive human emotions and may be influenced by them. These perceptions can affect the interactions between horses and their owners, riders or caretakers.

## Supplementary Information

Supplementary Information 1.

Supplementary Video 1.

## Data Availability

The datasets and R code generated and analyzed during the current study are available in the INRAE data repository from the following link: https://doi.org/10.57745/SLUKIO.
