# Supplementary material for: Horses discriminate human body odors between fear and joy contexts in a habituation-discrimination protocol
Source: Sci Rep. 2023 Feb 25;13:3285. doi: 10.1038/s41598-023-30119-8 (PMC9968287; doi:10.1038/s41598-023-30119-8)
Supplement: Supplementary file 1 — Supplementary Information 1. [file 41598_2023_30119_MOESM1_ESM.pdf]

Horses discriminate human body odors between fear and joy contexts in a habituation-discrimination protocol.

Plotine Jardat[1], Alexandra Destrez[2], Fabrice Damon[3], Zoé Menard--Peroy[1], Céline Parias[1], Philippe Barrière[4], Matthieu Keller[1], Ludovic Calandreau[1], Léa Lansade[1]

[1]CNRS, IFCE, INRAE, Université de Tours, PRC, F-37380, Nouzilly, France

[2]Developmental Ethology & Cognitive Psychology Laboratory, Centre des Sciences du Goût et de l'Alimentation, Institut Agro Dijon, CNRS, Université de Bourgogne-Franche-Comté, Inrae, Dijon, France
[3]Development of Olfactory Communication & Cognition Laboratory, Centre des Sciences du Goût et de l'Alimentation, Institut Agro Dijon, CNRS, Université de Bourgogne-Franche-Comté, Inrae, Dijon, France
[4]UEPAO, INRAE F-37380, Nouzilly, France

Supplementary Information

<u>Table S1</u>: Model selection results. Bold text highlights the models that were selected in the ANOVA because they were significantly different from the null model and minimized the AIC by more than two points. When two models satisfied these criteria, the most parsimonious model was retained. The variables of interest were the sample (A_1 or A_2 in the habituation phase and A_3 or B in the discrimination phase) and the group (joy for $A_{1\text{-}3}$ and fear for B, or vice versa), along with the side of the nostril for the number of nostril dilations.

Response variable (y)	Model type	Family	Phase	Formula	χ^2	AIC	DF	p value
Duration sniffing the odor	GLMM n=25*2 odors	Poisson	Habituation	Sniffing duration ~ 1		421.03		
				Sniffing duration ~ Sample	18.51	404.51	1	<0.0001
				Sniffing duration ~ Sample + Group	0.67	405.84	1	0.413
				**Sniffing duration ~ Sample * Group**	**10.41**	**397.43**	**1**	**0.0013**
			Discrimination	Sniffing duration ~ 1		370.55		
				**Sniffing duration ~ Sample**	**11.66**	**360.89**	**1**	**0.0006**
				Sniffing duration ~ Sample + Group	0.04	362.85	1	0.8334
				Sniffing duration ~ Sample * Group	3.71	361.14	1	0.0542
Number of nostril dilations	GLMM n=25*6 odors	Poisson	Habituation	Dilations ~ 1		425.62		
				Dilations ~ Side	9.99	417.64	1	0.0016
				**Dilations ~ Side + Sample**	**5.97**	**413.67**	**1**	**0.015**
				Dilations ~ Side * Sample	1.65	414.02	1	0.20
				Dilations ~ Side * Sample + Group	0.84	415.18	1	0.36
				Dilations ~ Side * Sample * Group	4.03	417.16	3	0.26
			Discrimination	Dilations ~ 1		351.14		
				Dilations ~ Side	0.01	353.13	1	0.91
				Dilations ~ Side + Sample	13.67	341.46	1	0.0002
				**Dilations ~ Side * Sample**	**11.3**	**332.16**	**1**	**0.0008**
				Dilations ~ Side * Sample + Group	0.01	334.14	1	0.90
				Dilations ~ Side * Sample * Group	8.05	332.09	3	0.045
